# Clinicopathological Characteristics, Prognosis, and Correlated Tumor Cell Function of Tropomodulin-3 in Pancreatic Adenocarcinoma

**DOI:** 10.2174/1386207326666230810142646

**Published:** 2024-04-26

**Authors:** Bin Zhong, Dan-Dan Ma, Tao Zhang, Qi Gong, Yi Dong, Jian-Xin Zhang, Zhong-Hu Li, Wei-Dong Jin

**Affiliations:** 1 The First School of Clinical Medicine, Southern Medical University, Guangzhou, 510515, China;; 2 Department of General Surgery, General Hospital of Central Theater Command, Wuhan, 430070, China

**Keywords:** TMOD3, pancreatic adenocarcinoma, prognosis, tissue microarray, TPM, PAAD patients

## Abstract

**Background:**

Pancreatic adenocarcinoma (PAAD) is a frequent malignant tumor with a high mortality rate. Searching for novel biomarkers that can influence its prognosis may help patients. It has been shown that tropomodulin-3 (TMOD3) may influence tumor progression, but its role in pancreatic cancer is not clear. We aimed to explore the expression and prognostic value of TMOD3 in PAAD.

**Methods:**

We used bioinformatics analysis to analyze the relationship between TMOD3 expression and clinicopathological features and prognosis and verified it with clinical data from tissue microarray. We also conducted *in vitro* cell experiments to explore the effects of TMOD3 on the function of PAAD cells.

**Results:**

TMOD3 expression was found to be significantly higher in PAAD tissues than in matched paracancerous tissues (*P <* 0.05). Meanwhile, high TMOD3 expression was associated with significantly poorer overall survival (*P <* 0.05). Analysis of relevant clinicopathological characteristics data obtained from TCGA showed that high TMOD3 expression correlated with age, TNM stage, N stage, and M stage (*P <* 0.05). Analysis of correlation data obtained from tissue microarrays showed that high TMOD3 expression was associated with lymph node invasion, nerve invasion, macrovascular invasion, and TNM stage (*P <* 0.05). In addition, siRNA knockdown of TMOD3 significantly reduced the migration and invasion of PAAD cells.

**Conclusion:**

Our study shows that TMOD3 may be associated with the progression of PAAD cells, and that it is an independent risk factor for poor pathological features and prognosis of PAAD. It may be helpful as a prognostic indicator of clinical outcomes in PAAD patients.

## 1 INTRODUCTION

Pancreatic adenocarcinoma (PAAD) is a malignancy with an inferior prognosis. Although many advances have been made in treating pancreatic cancer in recent years, the 5-year survival for PAAD is still only 8% [1, 2]. Most patients with PAAD are primarily asymptomatic in the early phase, and by the time they seek medical attention, most patients already find it difficult to intervene surgically [3-5]. In addition, due to the tumor's intrinsic chemotherapy and immune resistance, other therapeutic strategies, such as combined chemical therapy, molecularly targeted drugs, and immune checkpoint blockade, are uncertain for improving the prognosis [6-8]. Finding novel biomarkers may, therefore, have a meaningful impact on the early diagnosis and treatment of pancreatic cancer.

Tropomodulin (TMOD) family consists of four isoforms, tropomodulin-1, tropomodulin-2, tropomodulin-3, and tropomodulin-4 (TMOD1, TMOD2, TMOD3, and TMOD4), which are members of the capping protein family [9]. TMOD3 is unique in the TMOD family in its ability to cover the tropomyosin (TPM)-fibros(F)-actin and simple F-actin tips and selectively regulate the specialized TPM-F-actin network. It binds to actin and is regulated by Akt2 phosphorylation. These properties allow TMOD3 to potentially lead to the ability of tumor cells to acquire aggressive and metastatic properties, playing a vital role in the development of tumor invasiveness and migration [9]. Previous studies have indicated TMOD3 to have an affinity with the progression of various tumors [10-13]. However, the role of pancreatic cancer is unclear.

Therefore, we speculated that TMOD3 might be an independent predictor of poor PAAD outcomes and may be associated with the progression of PAAD based on previous findings. This study aimed to investigate the effect of TMOD3 on pancreatic cancer cell function, its expression in pancreatic cancer tissues, and its relationship with the clinicopathological features and prognostic value of PAAD patients.

## 2 MATERIALS AND METHODS

### 2.1 Bioinformatics Database Analysis

The TCGA (The cancer genome atlas, https://portal.gdc.cancer.gov/) was launched in 2006 by the National Cancer Institute (NCI) and the National Human Genome Research Institute (NHGRI) to collect clinical data on a wide range of human cancers, including subtypes of tumors [14]. We extracted information about the gene expression of TMOD3 in pancreatic cancer from this database and matched it to the clinicopathological features of pancreatic cancer patients through the R software. According to the median TMOD3 expression level, we divided the gene expression data into high and low groups.

We also downloaded the dataset GSE71729, which contains both normal and primary pancreatic cancer populations, from the GEO database [15] (https://www.ncbi.nlm.nih.gov/geo/) to compare the expression of TMOD3 in the two populations. We analyzed the gene expression of TMOD3 in various human cancers using the GEPIA (Gene Expression Profile Interactive Analysis, http://gepia.cancer-pku.cn/) tool [16] and matched it with paracancerous tissues for analysis. Metascape version 2021 (http://metascape.org/gp/index.html#/main/step1) was used for enrichment analysis of the TMOD3 interacting proteins [17]. GO, KEGG pathway, and Reactome gene sets were constructed to determine the biological functions related to the target genes.

### 2.2 Cell Lines and Cell Culture

The pancreatic cancer cell line, SW1990, was purchased from the Type Culture Collection of the Chinese Academy of Sciences. The culture condition included Dulbecco’s modified Eagle’s medium (DMEM; Gibco, USA) supplemented with 10% fetal bovine serum (Gibco, USA), 100 U/ mL penicillin, and 100 μg/mL streptomycin in a moist incubator. The whole cells and subcultures were cultured at a uniform temperature (37°C) in a 5% CO_2_ cultivation box.

### 2.3 RNA Transfection

Six-well plates were used for cell transfection. The synthesized pcDNA3.1-TMOD3 (TMOD3) and the empty plasmid pcDNA3.1 (Control) were purchased from the MiaoLing Plasmid Sharing Platform. The siRNA targeting TMOD3 (siRNA-TMOD3) was designed, and the following array was GAGCAUAUUAAUGAAAAGUGC. Meanwhile, the scrambled siRNA (siRNA-control) was built as the comparative processing. The transfection into cells of SW1990 was performed using Lipofectamine 2000 (Thermo, USA). The transfection efficiency was confirmed by western blotting at 48h post-transfection.

### 2.4 Cell Counting Kit-8 (CCK-8) Assay

CCK-8 assay was used to determine the cell viability. The transfected cells were incubated in 96-well plates at a concentration of 5 x 10^3^ cells per well in a 100µl complete growth medium for 24 and 48 hours, respectively, using the cell counting kit-8 (CCK-8; Beyotime, China). Then, 10µl CCK-8 reagent was added to each well and incubated for 2 hours. The optical density at 450 nm was confirmed by employing a microplate reader (BioTek, Winooski, Vermont, USA). Three self-contained replicates were carried out on each CCK-8 trial group.

### 2.5 Wound Healing Assay

The wound-healing experiment was used to research the influence of TMOD3 on PAAD SW1990 cell migration. Transfected cells (5 × 10^5^ cells/well) were seeded into 6-well plates with a fresh medium containing 10% FBS. The cell monolayer was scratched with a 10 µl sterile plastic pipette tip to generate a linear scratch wound. The wound was flushed with PBS three times and shot at 0 and 48 hours under an upside-down light microscope (magnification × 200; Olympus Corporation). The wound-healing rate was computed using the following equation: wound healing rate (%) = [(breadth at 0h - breadth at 48h) / (breadth at 0 h)] × 100%.

### 2.6 Transwell Invasion Assay

The cell invasion capacity was examined using a transwell assay. Cell suspensions (100µl, 5×10^4^ cells in serum-free DMEM medium) were added to the upper chamber containing an 8μm polycarbonate filter (Millipore, USA) that was pre-coated with 100µl diluted Matrigel (BD, USA). The lower chamber was filled with DMEM medium involving 10% FBS. After incubation at 37°C with 5% CO_2_ for 24h, the non-migrated cells at the top surface were removed. The invading cells on the lower surface were fixed in 4% paraformaldehyde for 15 min and stained in 0.1% crystal violet for 20 min. Images and cells counted were captured under an inverted microscope (magnification, × 200; Olympus Corporation).

### 2.7 Flow Cytometric Analysis

Flow cytometry (BD Biosciences, USA) was applied to test the apoptosis of SW1990 incubated with Annexin V-fluorescein isothiocyanate (FITC)/propidium iodide (PI) detection kit (BD, USA). FACS Calibur flow cytometer (BD, USA) was used to analyze the percentage of apoptotic cells.

### 2.8 Tissue Microarray and Immunohistochemistry

PAAD tissue microarrays (TMA) were purchased from Shanghai Outdo Biotech Co., LTD. TMA consisted of 105 PAAD tumor tissues and 28 matched paracancerous tissues. The biological samples experiment was approved by the General Hospital of Central Theater Command Medical Ethics Committee. The immunohistochemical technique of PAAD TMA was employed using an immunohistochemical kit (EnVision™Flex+, CAT). Immunohistochemical staining was scored using the Aperio scanner (Aperio XT, Leica Microsystems GmbH, Amplomation). The TMA slides were incubated overnight at 4°C with rabbit polyclonal antibody against TMOD3 (Cat No: 14247-1-AP, 1:50, Proteintech, USA). After washing with phosphate-buffered saline (PBS), substrate-chromogen and peroxidase-labeled polymer were applied to visualize the protein staining. The visual immunoreactivity score (IRS) [18] was used to assess the expression of TMOD3 protein. The CaseViewer 2.4 software (3DHISTECH Ltd.) was used to read slices and analyze the TMA. The score criteria of staining intensity (SI) are listed as follows: none = 0, weak = 1, moderate = 2, and strong =3. The percentage of positive cells was as follows: negative = 0, <10% positive cells = 1, 10% ~ 50% positive cells = 2, 51% ~ 80% positive cells = 3, > 80% positive cells = 4. The final IRS was calculated by SI × percentage of positive cells. We divided the samples into low (IRS ≤ 4) or high expression (IRS > 4).

## 3 STATISTICAL ANALYSIS

All numerical data have been expressed as numbers and percentages, while quantitative data have been expressed as mean ± standard deviation. The Chi-square test or Mann-Whitney test, t-test, or Wilcoxon test have been employed for a comparison between the groups depending on the data. All p-values have been two-sided. Data have been statistically analyzed using the R software (version 4.1.1; R Foundation for Statistical Computing). Prognostic independent risk factors were evaluated using the Cox regression method. The statistical analyses of cellular functions were performed using GraphPad Prism Software 9.0. The significance level was set at 5%.

## 4 RESULTS

### 4.1 Analysis of TMOD3 Expression in the Bioinformatics Database

**Fig. (1):**
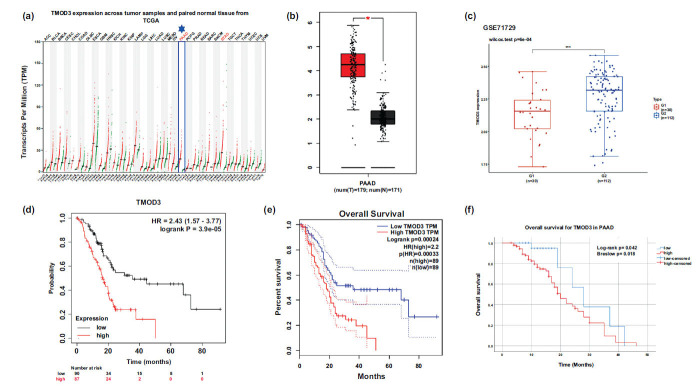
Expression of TMOD3 in multiple databases and prognostic analysis. (**a**) TMOD3 expression in pan-cancer, analyzed by GEPIA. (**b**) Comparison of TMOD3 expression in tumor and normal tissues by applying the GEPIA tool to identify remarkably improved expression by high log_2_FC values and percentage values above the threshold. *: *P <* 0.05. (**c**) TMOD3 expression in the pancreatic carcinoma group and the normal group in GSE71729; G1 for the normal group and G2 for the pancreatic carcinoma group. (**d**) Kaplan-Meier curve analysis of TMOD3 expression and prognostic value in GSE71729 (*P <* 0.05) was considered statistically significant. (**e**) Survival prognosis analysis on the basis of TMOD3 expression (from TCGA datasets). HR: hazard ratio. *P < 0.05. (**f**) The effect of TMOD3 protein expression on overall survival was analyzed by applying Kaplan-Meier analysis and log-rank. The expression level of TMOD3 was determined by immunohistochemical staining of PAAD tissue microarrays.

TMOD3 was only highly expressed in gastric adenocarcinoma (STAD) and pancreatic adenocarcinoma (PAAD) cancers (*p*<0.05). In contrast, TMOD3 was not differentially expressed in other cancers, such as adrenocortical carcinoma (ACC) and lung adenocarcinoma (LUAD) (Fig. **1a**). We also used the GEPIA tool to explore TMOD3's expression in PAAD tissues and matched cancer-adjacent tissues. The result showed that TMOD3 was increased markedly in PAAD tissue (*P <* 0.05) (Fig. **1b**). The GSE71729 dataset includes data from both pancreatic cancer patients and normal patients. Therefore, we divided normal patients into group G1 and patients with pancreatic cancer into group G2, and explored the difference in TMOD3 expression in the two groups. The results showed the expression of TMOD3 to be higher in G2 than in G1 (Fig. **1c**).

Statistical analysis revealed TMOD3 expression levels to be related to the following factors: age (p = 0.006), TNM stage (*p* <0.001), N stage (*p* = 0.017), and M stage (*p<*0.001). TMOD3 expression was not significantly associated with sex, grade, and T-stage (*p*>0.05) (Table **1**). Multi-variable analysis with the Cox proportional hazards model showed the following variables to be independent risk factors for OS: TMOD3 (HR: 1.746; 95%CI: 1.019-2.992; *P <* 0.001), N stage (HR: 1.128; 95% CI: 1.060-1.201; *P* = 0.004) and age (HR: 1.032; 95%CI: 1.010-1.054; *P* = 0.004) (Table **2**).

We used multiple databases to explore the relationship between TMOD3 expression and pancreatic cancer prognosis. The prognostic value of TMOD3 in GSE71729 was explored using Kaplan-Meier analysis. We noted pancreatic ductal adenocarcinoma patients with elevated TMOD3 levels to be associated with a decrease in overall survival (*P <* 0.01) (Fig. **1d**). We then analyzed data from 178 PAAD patients extracted from the TCGA. The expression of TMOD3 in these patients was divided into high and low groups. The results showed low TMOD3 expression to be correlated with a better prognosis (*P* <0.01; Fig. **1e**).

### 4.2 Functional Enrichment Analysis Of Candidate TMOD3 Interactive Proteins

Enrichment analysis of the pathway allowed us to identify the biological processes and functions in which 164 interacting proteins may be involved (Supplementary Material **1**). In the Metascape analysis, the target genes of TMOD3 were found to be involved in GO biological processes, including ‘actin filament-based process’, ‘actin filament-based movement’, ‘cell division’, ‘regulation of cell morphogenesis’, *etc*., and involved in enrichment in the following KEGG approaches: ‘regulation of actin cytoskeleton’ and ‘bacterial invasion of epithelial cells’ signaling pathway’. Metascape analysis was applied to recognize the relevant Reactome gene sets, comprising ‘membrane trafficking’, ‘gap junction degradation’, *etc*. (Supplementary Material **2**). PPI network analyses were further performed using Cytoscape software to study the relationship between the 164 overlapping target genes. The MCC algorithm was used to identify dense networks and to connect each gene with round color frames to represent the rank score. The higher the ranking, the redder the color in the oval box (Supplementary Material **3**).

### 4.3 *Vitro* Cell Experiment

**Fig. (2):**
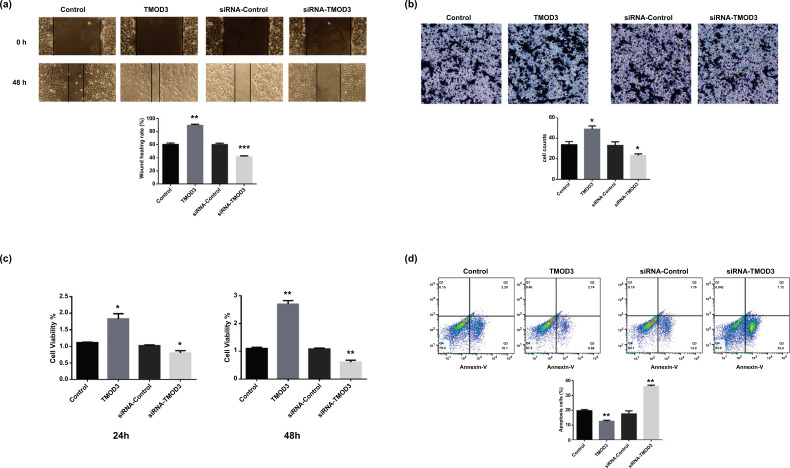
Effect of TMOD3 on proliferation, migration, invasion, and apoptosis of PAAD cells. (**a**) Wound healing assay. Overexpression of TMOD3 promoted migration of SW1990 cells, whereas knockdown of TMOD3 inhibited migration of SW1990 cells. (**b**) Transwell invasion assay. Overexpression of TMOD3 promoted SW1990 cells’ invasion and knockdown of TMOD3 suppressed SW1990 cells’ invasion. (**c**) CCK8 assay. Overexpression of TMOD3 promoted SW1990 cells’ proliferation and knockdown of TMOD3 suppressed SW1990 cells’ proliferation. (**d**) Flow cytometric analysis. TMOD3 had a significant influence on the apoptosis of SW1990 cells. *: *P <* 0.05; **: *P <* 0.01; ***: *P <* 0.001.

We constructed a TMOD3 overexpression and silencing model to investigate whether TMOD3 affects the invasion and migration of PAAD cells. Through *in vitro* cellular assays, we aimed to assess the effect of TMOD3 on the biological function of pancreatic cancer cells. Migration of SW1990 cells was markedly inhibited 48 hours after transfection compared to the control and siRNA-control groups. Overexpression of TMOD3 promoted PAAD cell migration based on the results in which the TMOD3 group wound healing rate was faster than the control group and the siRNA-control group (Fig. **2a**).

The Transwell invasion experiment was used to assess the effect of TMOD3 on the invasion ability of SW1990 cells. Cell invasion was markedly lowered in the siRNA-TMOD3 group compared to the siRNA-control group, while the TMOD3 group remarkably exhibited promoted cell invasion compared to the control group. Meanwhile, there was no statistical difference between the control and siRNA-control groups. This suggests that TMOD3 may promote PAAD cells’ invasion (Fig. **2b**).

The results of the CCK-8 assay showed that overexpression of TMOD3 significantly promoted SW1990 cell viability compared to the control because the OD value of the TMOD3 group was higher than the control group. Similarly, siRNA-TMOD3 significantly suppressed SW1990 cell viability compared to siRNA-control. There was no difference between the control and siRNA-control (Fig. **2c**).

We found the apoptosis cells of the TMOD3 group to be less than the control group, and the apoptosis cells of the siRNA-TMOD3 group were more than the siRNA-control group (Fig. **2d**). There was no difference between the control and siRNA-control. Thus, we concluded that TMOD3 might affect apoptosis in SW1990 cells.

### 4.4 Immunohistochemistry

**Fig. (3):**
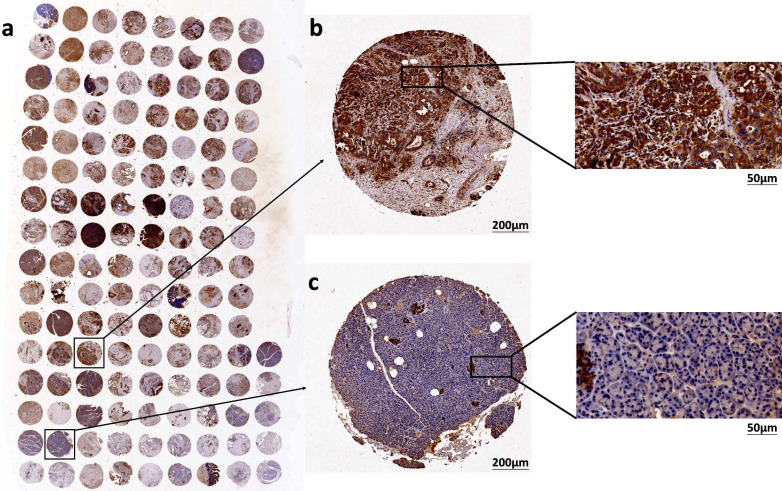
(**a**) Full view of immunohistochemical staining of TMOD3 in the TMA cohort with 105 pancreatic cancer tissues and 28 paracancerous tissues. (**b**) Micrographs of TMOD3 immunohistochemically stained as positive. (**c**) Micrographs of negative TMOD3 immunohistochemical staining.

Immunohistochemical techniques were applied to determine the expression of TMOD3 in PAAD and matched cancer-adjacent tissues, which were classified into high and low groups according to IRS (Fig. **3**). The results showed that TMOD3 showed high expression in 91 pancreatic cancer tissue samples (79.14%) and low expression in the remaining samples (20.86%). However, in matched cancer-adjacent tissues, TMOD3 showed high expression in 6 samples (33.33%) and low expression in 12 samples (66.67%). TMOD3 expression was significantly increased in PAAD tissues compared to matched paracancerous tissue samples (*P <* 0.001; Table **3**).

### 4.5 TMOD3 Expression Levels Correlate with Clinicopathological Features and Prognosis in Clinical Samples

The results of the statistical analysis showed that the expression level of TMOD3 was associated with lymph node invasion (*P*=0.039), nerve invasion (*P*=0.049), macrovascular invasion (*P*=0.025), and TNM stage (*P*=0.040). The expression of TMOD3 was not statistically correlated to other clinical pathological features (Table **4**).

We performed Log-rank tests and plotted survival curves using the Kaplan-Meier method. The results showed the overall survival to be significantly lower in the high TMOD3 expression group compared to the low expression group (Fig. **1f**). This demonstrated a possible relationship between high TMOD3 expression and unfavorable prognostication in PAAD, and suggested that TMOD3 may be an independent risk factor for the prognosis of PAAD.

## 5 DISCUSSION

PAAD is one of the more malignant cancers, and its mortality rate remains high in more developed countries [19]. Due to the highly aggressive nature of pancreatic cancer, the discovery of new tumor-specific targets that inhibit pancreatic cancer progression is key to prolonging tumor-free survival and overall survival after surgical treatment of pancreatic cancer. Many new therapeutic approaches have also been presented in recent years, such as targeted therapy that targets the pathophysiological processes involved in PAAD [20]. However, in pancreatic cancer patients with postoperative or advanced inoperable pancreatic cancer, it is equally vital to inhibit the progression of PAAD for prolonging patient survival. TMOD3 selectively regulates the specialized TPM-F-actin network and is regulated by Akt2 phosphorylation upon binding to actin to accelerate tumor metastasis [9, 21]. Therefore, we suggest that TMOD3 may play an essential role in the progression of PAAD; however, there are no detailed studies on the role of TMOD3 in pancreatic cancer and its effect on PAAD migration and invasion.

In this research, we employed a multi-database for bioinformatics analysis to explore the differential expression of TMOD3 in patients and tissues of pan- and pancreatic cancer and explored the correlation between TMOD3 and the prognosis of PAAD. We also performed validation using *in vitro* cellular assays and TMA. Our validation on multiple fronts supports the hypothesis that TMOD3 may promote PAAD progression. First, TMOD3 expression was significantly higher in PAAD tissues than in matched paracancerous tissues. Also, TMOD3 expression was significantly higher in PAAD patients than in matched normal populations. Secondly, analysis of clinical data obtained from TCGA showed that high expression of TMOD3 was closely associated with N and M stages. Analysis of clinical data from TMA showed that high TMOD3 expression was associated with lymph node invasion, nerve invasion, macrovascular invasion, and TNM stage. Both the results showed TMOD3 to be associated with invasion and metastasis in PAAD, suggesting that high TMOD3 expression may be closely associated with PAAD progression. Finally, we also showed by employing *ex vivo* experiments that the deletion of TMOD3 restrained the transportation and invasion ability of SW1990 cells. Interestingly, we also found that TMOD3 may affect the proliferation and apoptosis of PAAD cell lines, although the CCK-8 assay only showed cell viability, and further confirmation of this potential would require cell cycle, clone formation, and EdU assay to examine proliferation.

To explore the underlying mechanism by which TMOD3 affects the progression of PAAD, we applied bioinformatic techniques to identify the interaction partners of TMOD3 and enrichment analysis of these genes. Enrichment analysis by Metascape showed that most of the interaction partners of TMOD3 are linked to the regulation of actin cytoskeleton and actin filament-based process. This is in line with previous studies reporting the effects of TMOD3 on actin [22, 23]. Since abnormal expression of genes regulating the actin cytoskeleton is closely related to the migration and invasion of tumors, targeted cancer therapy is of great significance [24-27]. Based on the available data, it has been found that the mechanism of TMOD3 for promoting cancer cell invasion and metastasis may be related to EGFR-PI3K-AKT and MAPK/ERK, and other related signaling pathways [28, 29]. Identifying the interacting partners of TMOD3 also paves the way for subsequent studies on the related pathway mechanisms. Of course, our subsequent studies need to explore the upstream signaling pathways.

Although previous studies have explored the molecular mechanisms associated with TMOD3 invasion and metastasis in hepatocellular carcinoma, non-small cell lung cancer, and metastatic cell carcinoma of the bladder [10, 11, 13], the relationship between TMOD3 and PAAD invasion and metastasis has not been explored. Other researches in multi-database have concentrated only on selecting hub genes [30-32], but they have not concretely analyzed the molecular mechanisms underlying core gene expression, prognosis, and role. This study has addressed this issue by observing TMOD3 expression in tissue samples, analyzing clinicopathological characterization data, exploring molecular mechanisms, and validating cellular experiments. TMOD3 is a key gene affecting the progression of pancreatic carcinoma. It may be an independent predictor of poor outcomes.

Only a single pancreatic cancer cell line was used for this study, which was established in 1978 from splenic metastases from a patient with stage II pancreatic adenocarcinoma of the exocrine gland of the pancreas. Since we have mainly explored the invasive and metastatic properties of TMOD3 and pancreatic cancer, it is feasible to use this cell line for functional experiments with the relevant cells. However, the number of PAAD cell lines should still be increased to perform more comprehensive functional experiments *in vitro*, which is a shortcoming of this study. Meanwhile, in exploring the relationship between TMOD3 expression levels and clinicopathological features, analysis using only a tiny amount of clinicopathological data from the TCGA database may be unreliable. To compensate for this, we have used clinical data from TMA for analytical validation, thus strengthening the clinical value of TMOD3 and the article's credibility. Nevertheless, additional cohort studies on the association between TMOD3 and the prognosis and adverse pathological features of PAAD patients are needed to compensate for the bias arising from single-center data. Furthermore, we have only demonstrated the effect of TMOD3 on PAAD cell invasion and migration in terms of cellular experiments, and further validation from other perspectives (*e.g*., animal experiments) may be needed. In addition to exploring reliable biomarkers for early diagnosis of pancreatic cancer, it is essential to identify patient groups that would benefit from targeted therapy. Our findings may contribute to the research and clinical application of postoperative adjuvant therapy for patients with pancreatic cancer.

## CONCLUSION

In conclusion, TMOD3 may be an independent predictor of poor prognosis in PAAD and associated with the progression of PAAD. TMOD3 can potentially be used in the prognostic assessment of PAAD, and after further studies, with the development of the targeted drug, the survival of pancreatic cancer patients may be improved.

## AUTHORS’ CONTRIBUTIONS

ZB, ZH, DD, and WD conceived the idea for the study; ZH, DY, and ZT performed the experiments; ZB, GQ, and JX analyzed the data; ZB and DD wrote the manuscript. All authors have read and approved the final version of the manuscript.

## Figures and Tables

**Table 1:** Correlation between TMOD3 expression and clinical pathological features in PAAD patients.

**Clinicopathological features**	**Variables**	**TMOD3 expression**			
		**Low (n=86)**	**High (n=87)**	***χ2***	***p***
Age (years)	-			7.419	**0.006**
Sex	≤60 >60	19 67	36 51	0.140	0.708
Grade	Male Female G1/G2 G3/G4	46 40 65 21	49 38 58 29	1.673	0.196
TNM stage	-			14.357	**<0.001**
	I/II	79	60		
	III/IV	4	3		
T stage				0.702	0.402
	T1/T2	17	13		
	T3/T4	69	74		
N stage	-			5.744	**0.017**
	N0 N1	32 54	18 69		
M stage				17.037	**<0.001**
	M0 M1	81 5	61 26		

**Table 2:** Univariate and multivariate analyses of OS in pancreatic carcinoma by Cox regression analysis.

**Variables**	**Univariate Analysis**			**Multivariate Analysis**		
	**HR**	**95% CI**	***P***	**HR**	**95% CI**	***P***
Age (>60 vs. ≤60)	1.026	1.005-1.050	0.018	1.032	1.010-1.054	0.004
Gender (female vs. male)	0.781	0.513-1.191	0.247	-	-	-
Grade (G4/G3/G2/G1)	1.338	0.998-1.795	0.050	1.157	0.846-1.583	0.361
Stage (IV/III/ II /I)	1.078	0.855-1.359	0.314	-	-	-
T-stage (T4/T3/T2/T1)	1.490	0.923-2.405	0.102	-	-	-
N-stage (N1/N0)	1.111	1.051-1.174	0.004	1.128	1.060-1.201	0.004
M-stage (M1/M0)	1.031	0.598-1.778	0.991	-	-	-
TMOD3 expression(High/Low)	2.180	1.282-3.706	< 0.001	1.746	1.019-2.992	< 0.001

**Abbreviations:** OS: Overall Survival, HR: Hazard Ratio, CI: Confidence Interval.

**Table 3:** Comparison of TMOD3 expression levels in PAAD tissues and paired paracancerous tissues.

***TMOD3 Expression***					
**Tissue Type**	**N**	**Low**	**High**	***χ2***	***p***
Pancreatic cancer	115	24	91	16.53	< 0.001
Paracancerous tissues	18	12	6	-	-

**Abbreviations:** TMOD3: Tropomodulin-3; PAAD: pancreatic adenocarcinoma.

**Table 4:** Correlation between TMOD3 expression and clinical pathological features in TMA clinical data.

**Clinicopathological Features**	**Variables**	**TMOD3 Expression**	**-**	***χ2***	***p***
		**Low (n=24)**	**High (n=91)**		
Age (years)	**-**			0.027	0.869
Sex	≤60 >60	13 11	51 40	0.971	0.324
Tumor Location	Male Female Pancreatic head Pancreatic body/tail	4 20 20 4	24 67 75 16	0.011	0.916
Tumor Size (cm)	**-**			0.028	0.867
	≤2 >2	8 16	32 59	**-**	**-**
Lymph Node Invasion	**-**			4.263	0.039
	Yes	5	40	**-**	**-**
	No	19	51	**-**	**-**
Nerve Invasion				3.888	0.049
	Yes No	3 21	30 61	**-**	**-**
Macrovascular Invasion	**-**			5.038	0.025
	Yes No	5 19	42 49	**-**	**-**
Duodenal Invasion	**-**			0.051	0.822
	Yes	3	13	**-**	**-**
	No	21	78	**-**	**-**
Liver Metastasis				0.008	0.929
	Yes	3	12	**-**	**-**
	No	21	79	**-**	**-**
Tissue Differentiation	**-**			0.546	0.460
	Low	7	20	**-**	**-**
TNM stage	Middle/High	17	71	4.232	0.040
	I-II	17	43	**-**	**-**
	III-IV	7	48	**-**	**-**

## Data Availability

The datasets generated and/or analyzed during the current study are available in the TCGA (https://portal.gdc.cancer.gov/) and GEO (https://www.ncbi.nlm.nih.gov/geo/geo2r/?acc=GSE71729) repositories. The data on TMA clinical samples used and analyzed during the current study will be available from the corresponding author upon reasonable request.

## LIST OF ABBREVIATIONS

ACC: Adrenocortical Carcinoma
CCK-8: Cell Counting Kit-8
DMEM: Dulbecco's Modified Eagle Medium
F: Fibros
FBS: Fetal Bovine Serum
IHC: Immunohistochemistry
LUAD: Lung Adenocarcinoma
OD: Optical Density
OS: Overall Survival
PAAD: Pancreatic Cancer
PBS: Phosphate Buffer Saline
PPI: Protein-Protein Interaction
PRAD: Prostate Adenocarcinoma
STAD: Gastric Adenocarcinoma
TMA: Tissue Microarray
TMOD1: Tropomodulin-1
TMOD2: Tropomodulin-2
TMOD3: Tropomodulin-3
TMOD4: Tropomodulin-4
TPM: Tropomyosin
