# Supplementary material for: Clinicopathological Characteristics, Prognosis, and Correlated Tumor Cell Function of Tropomodulin-3 in Pancreatic Adenocarcinoma
Source: Comb Chem High Throughput Screen. 2024 Apr 26;27(7):1011–21. doi: 10.2174/1386207326666230810142646 (PMC11165712; doi:10.2174/1386207326666230810142646)
Supplement: Supplementary file 1 [file CCHTS-27-1011_SD1.pdf]

Supplementary Material

Clinicopathological Characteristics, Prognosis, and Correlated Tumor Cell Function of Tropomodulin-3 in Pancreatic Adenocarcinoma

Bin Zhong[1,#], Dan-Dan Ma[2,#], Tao Zhang[2], Qi Gong[2], Yi Dong[1], Jian-Xin Zhang[2], Zhong-Hu Li[2] and Wei-Dong Jin[2,*]

[1]The First School of Clinical Medicine, Southern Medical University, Guangzhou, 510515, China; [2]Department of General Surgery, General Hospital of Central Theater Command, Wuhan, 430070, China

Table SI. Gene Set Enrichment Analysis for TMOD3 target genes.

Term	Description	Count	Frequency,%	Log10(P)	Log10(q)
A, GO Biological Process					
GO:0030029	actin filament-based process	67	40.85	-59.09	-54.73
GO:0030048	actin filament-based movement	18	10.98	-19.36	-16.48
GO:0034330	cell junction organization	27	16.46	-14.22	-11.60
GO:0051301	cell division	23	14.02	-11.82	-9.34
GO:0022604	regulation of cell morphogenesis	17	10.37	-11.41	-8.94
GO:0051014	actin filament severing	6	3.66	-9.57	-7.56
GO:0006897	endocytosis	20	12.20	-8.98	-6.66
GO:0030031	cell projection assembly	19	11.59	-8.87	-6.55
GO:0051668	localization within membrane	20	12.20	-8.81	-6.55
GO:0051017	actin filament bundle assembly	11	6.71	-8.59	-6.28
GO:0000226	microtubule cytoskeleton organization	19	11.59	-8.28	-6.00
GO:0010256	endomembrane system organization	17	10.37	-7.75	-5.49
B, KEGG Pathway					
hsa04810	Regulation of actin cytoskeleton	24	14.63	-23.34	-20.32
hsa05100	Bacterial invasion of epithelial cells	15	9.15	-17.70	-14.90
C, Reactome Gene Sets					
R-HSA-9716542	Signaling by Rho GTPases, Miro GTPases and RHOBTB3	41	25.00	-28.07	-24.89
R-HSA-199991	Membrane Trafficking	32	19.51	-20.21	-17.29
R-HSA-190873	Gap junction degradation	8	4.88	-15.27	-12.59
R-HSA-9659379	Sensory processing of sound	12	7.32	-13.64	-11.05
R-HSA-5627123	RHO GTPases activate PAKs	7	4.27	-10.20	-7.80

KEGG: Kyoto Encyclopedia of Genes and Genomes; q: false positive rate.

ACTA2

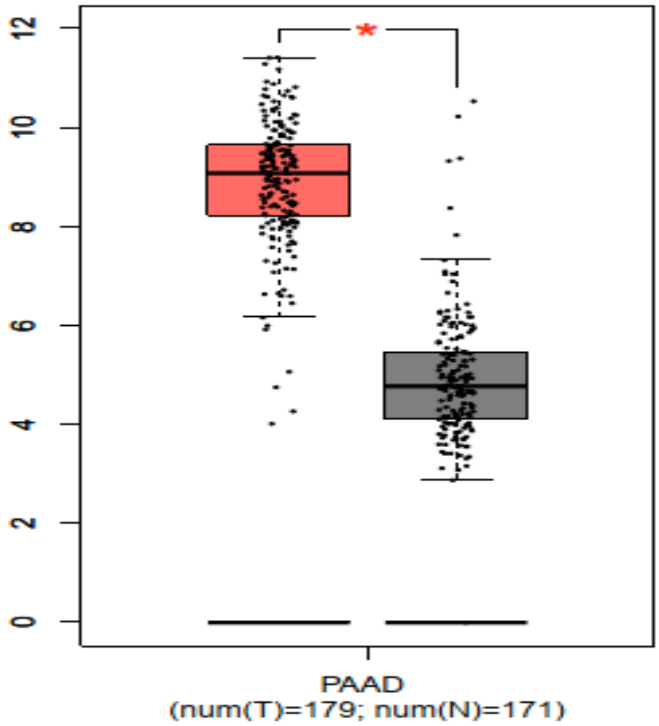
0
2
4
6
8
10
12
*
PAAD
(num(T)=179; num(N)=171)

ACTB

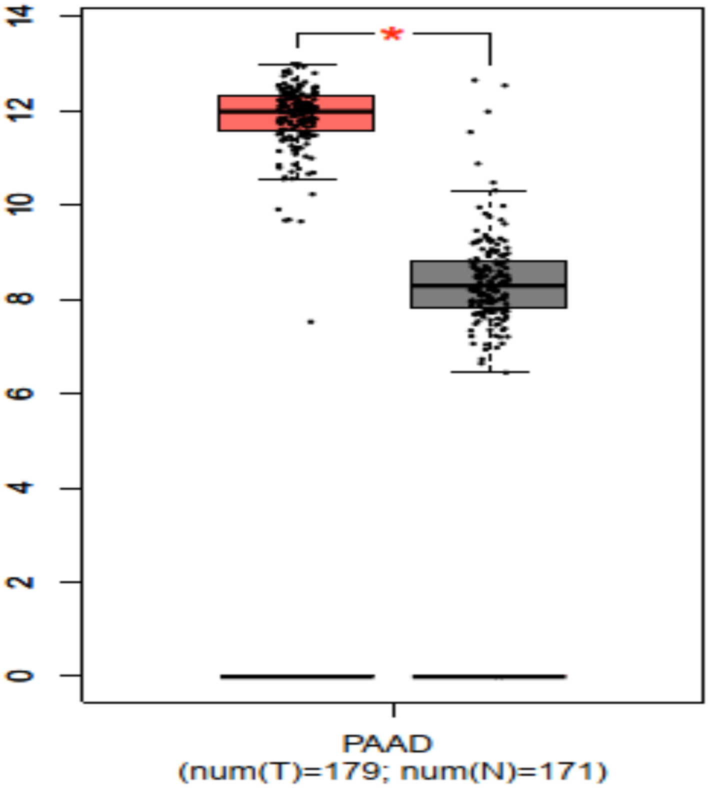
0
2
4
6
8
10
12
14
*
PAAD
(num(T)=179; num(N)=171)

ACTG1

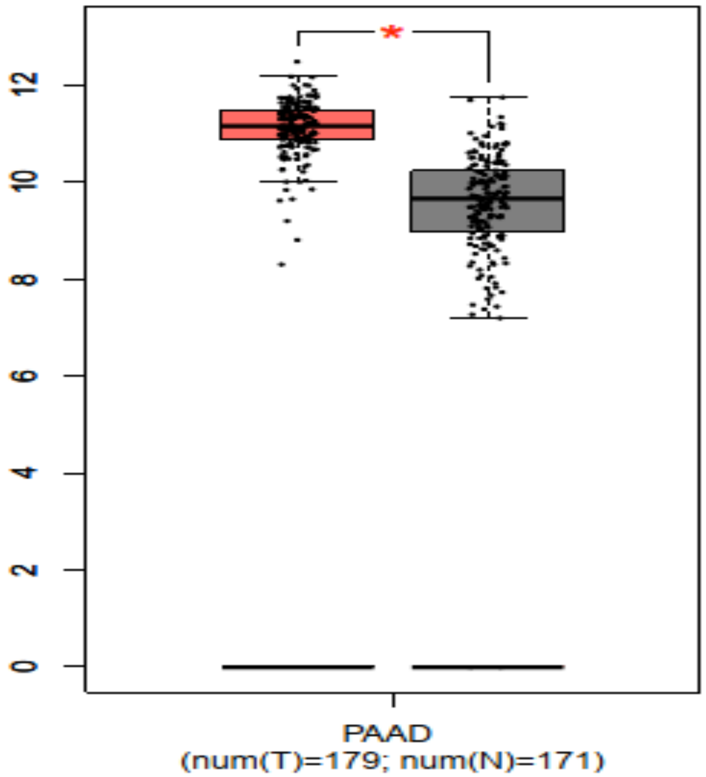
*
12
10
8
6
4
2
0
PAAD
(num(T)=179; num(N)=171)

ACTN4

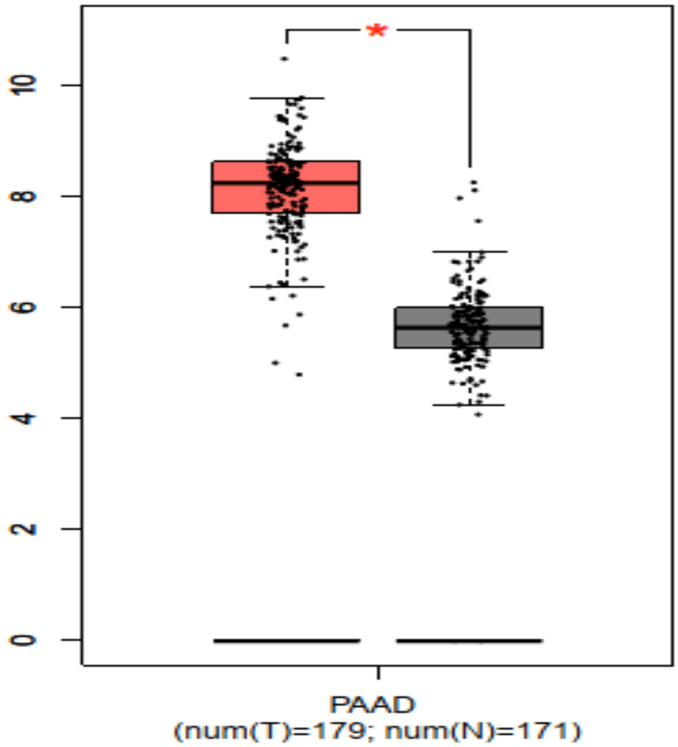
*
10
8
6
4
2
0
PAAD
(num(T)=179; num(N)=171)

ACTR2

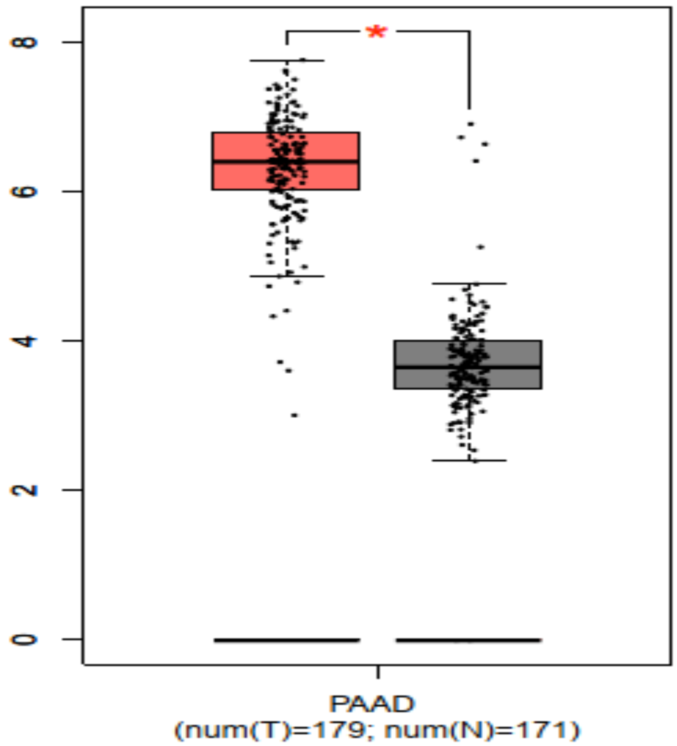

ANLN

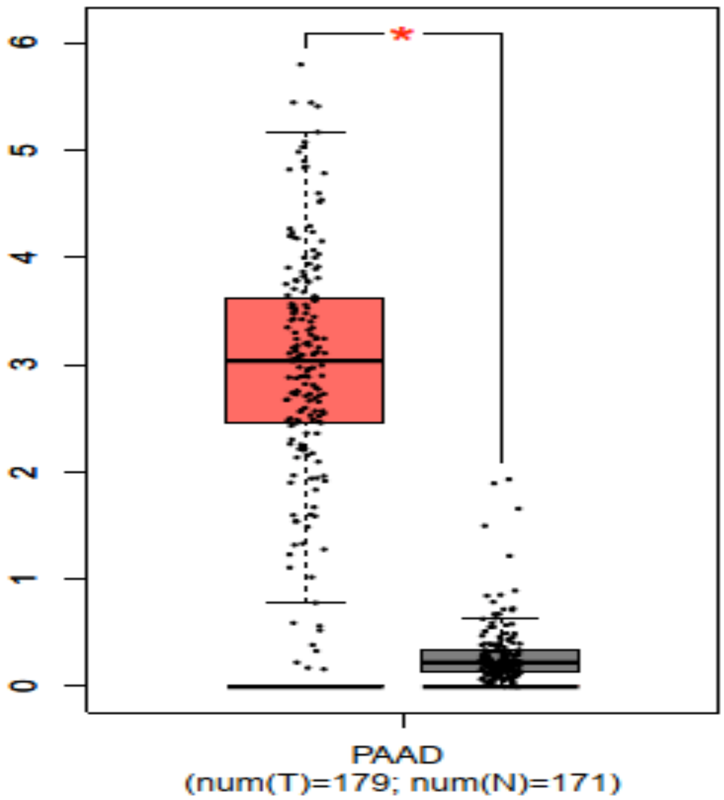

ARPC1B

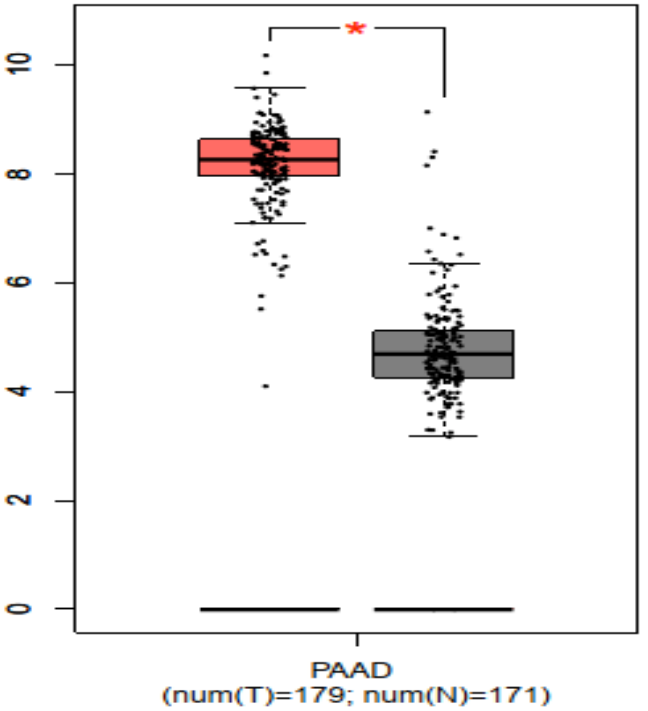

ARPC2

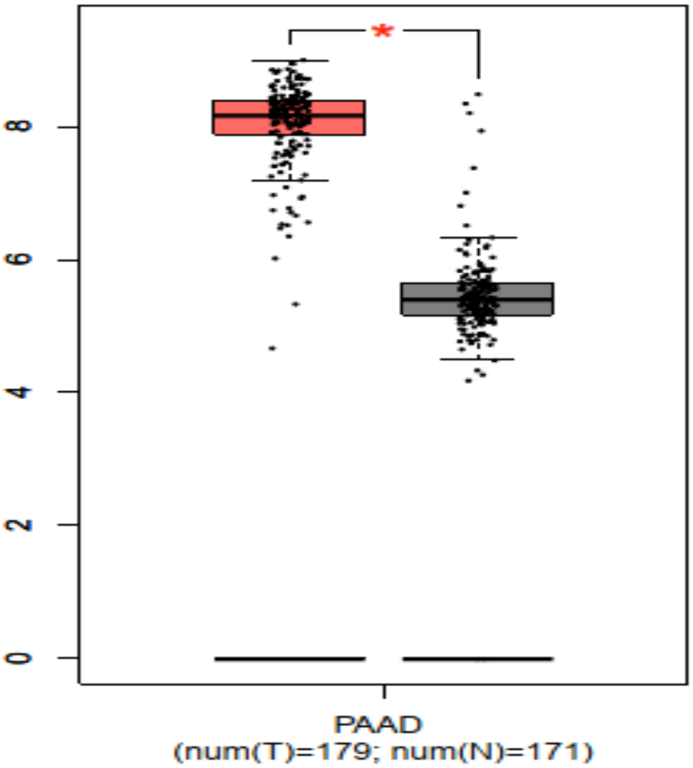

ARPC3

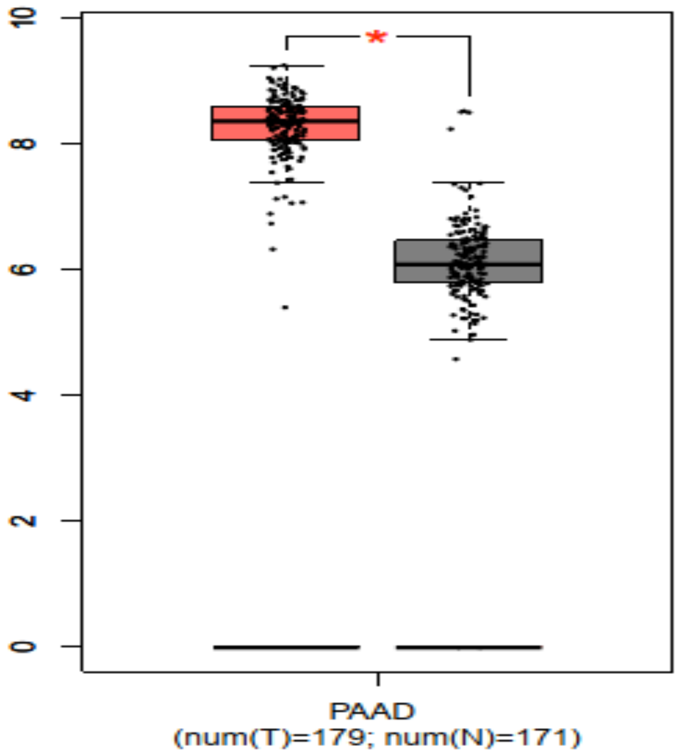

ARPC4

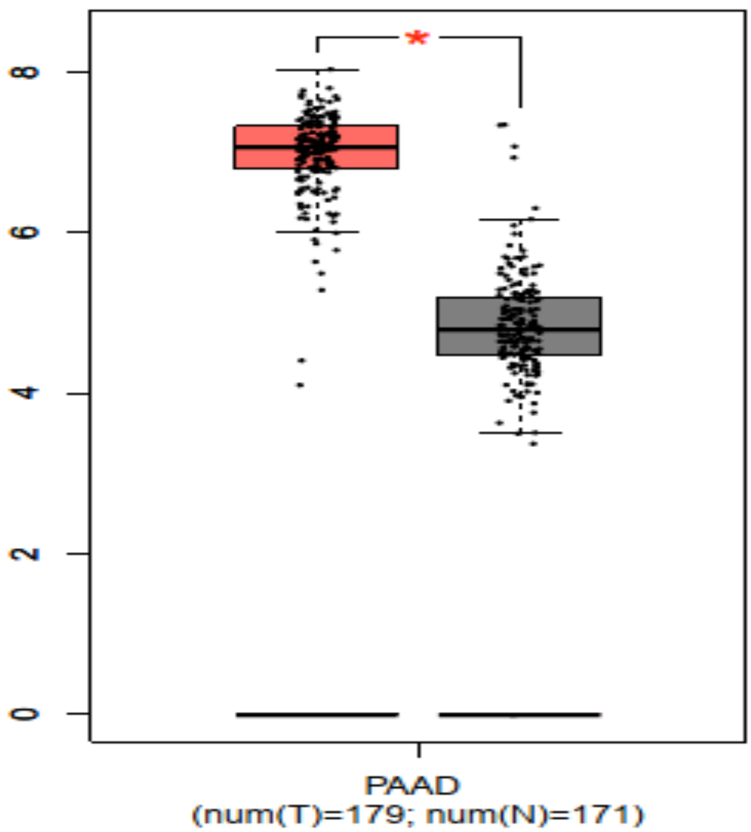

ARPC5L

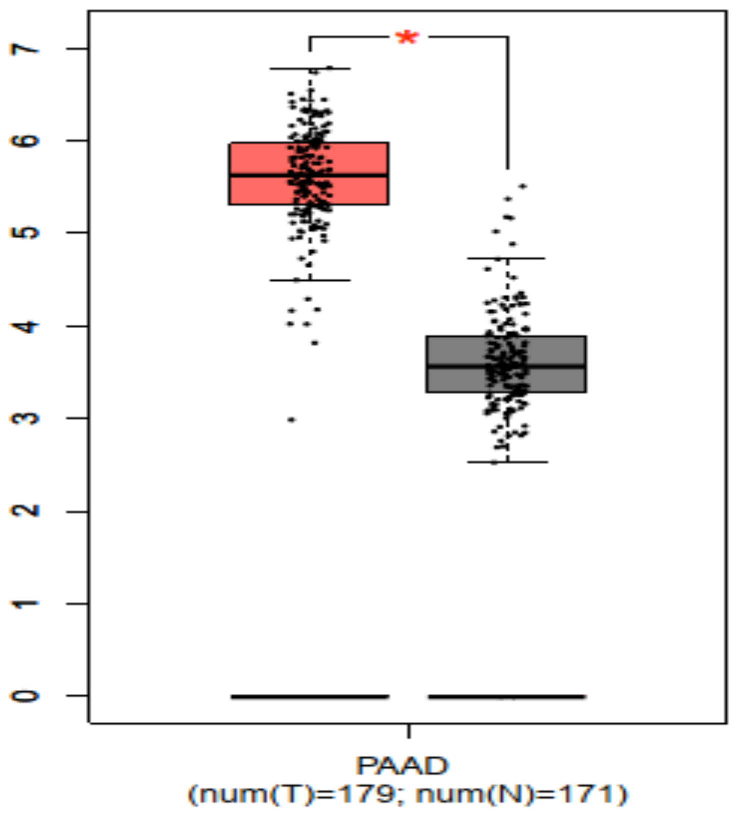

CAPZA1

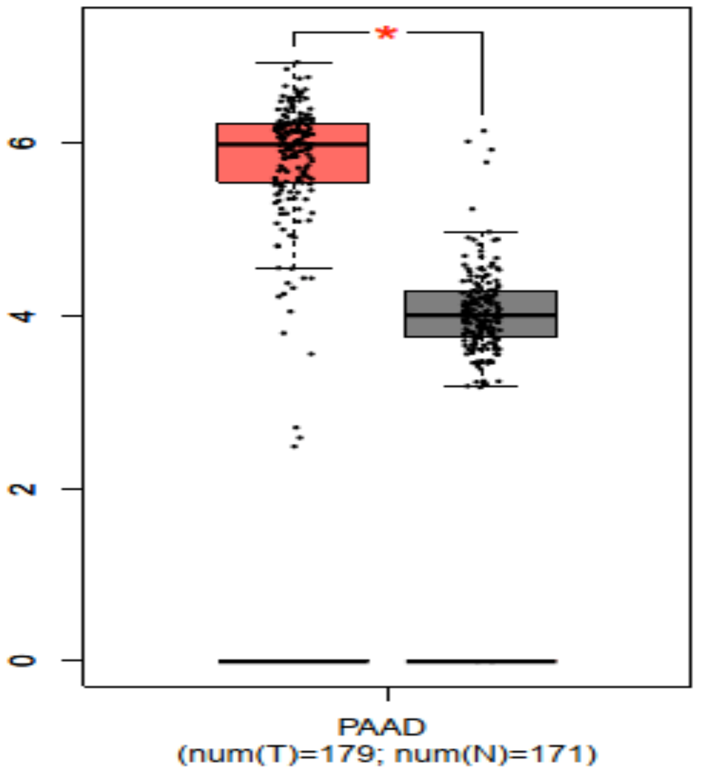

CAPZA2

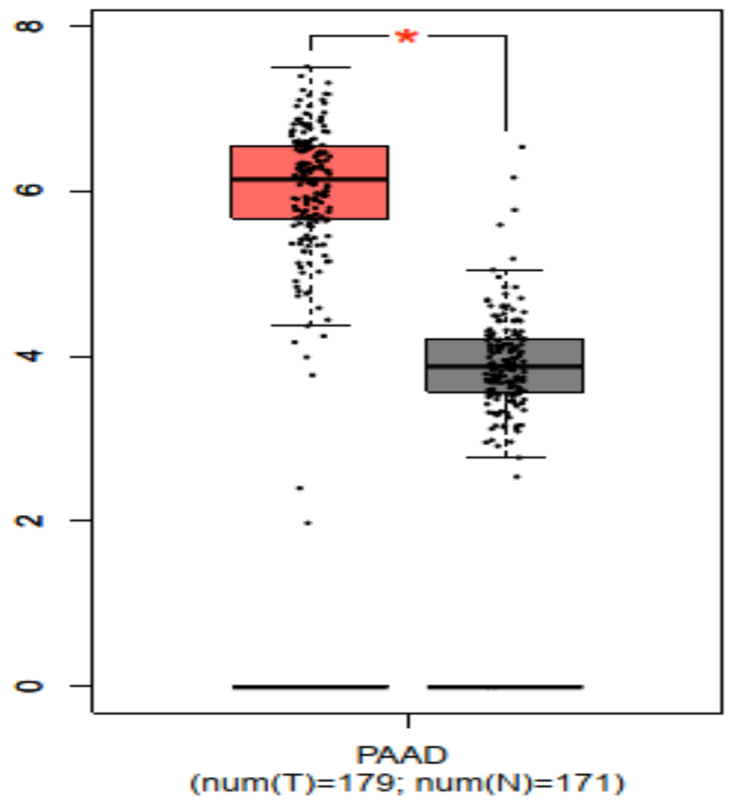

CAPZB

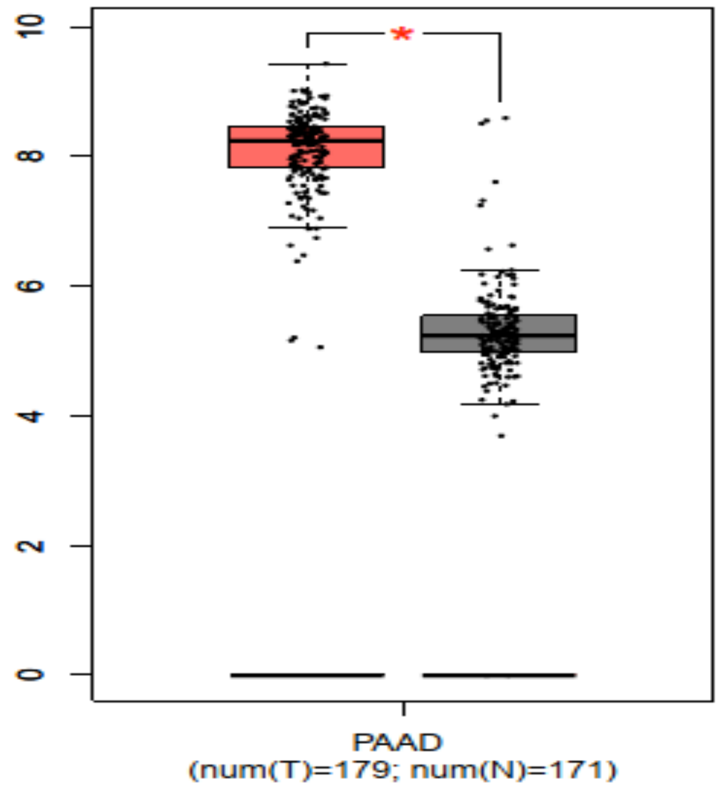

CDC42

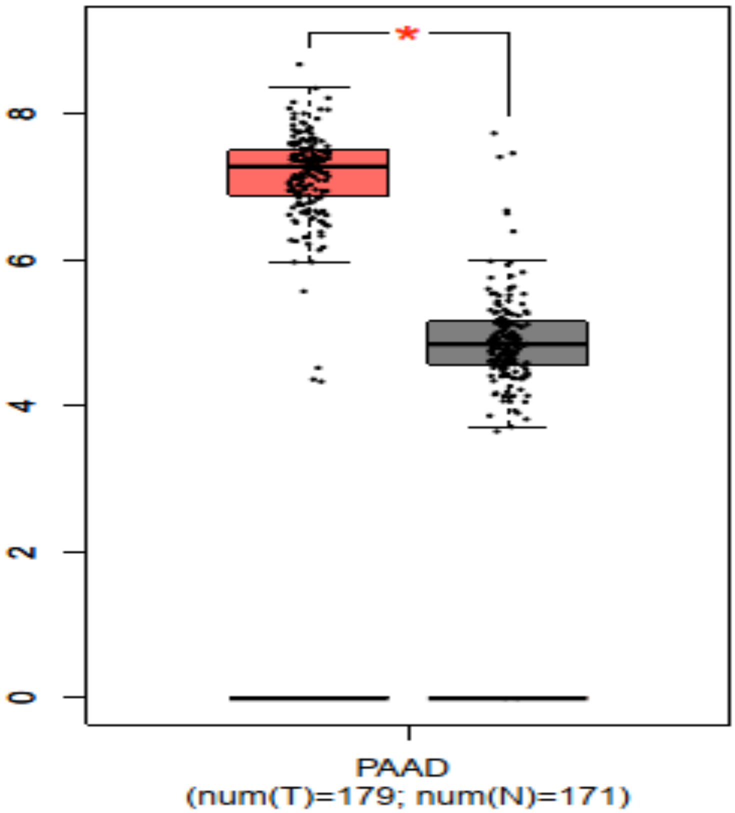

CFL1

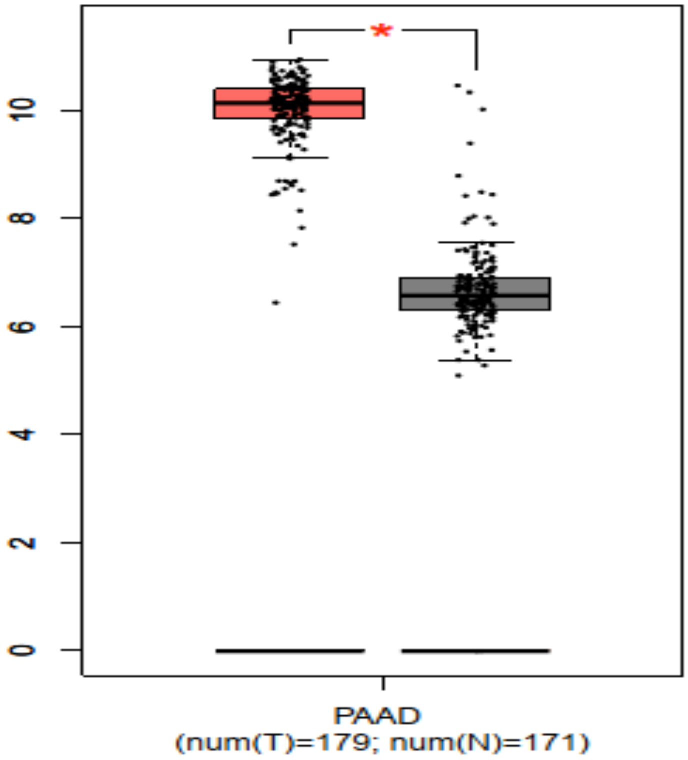

CORO1C

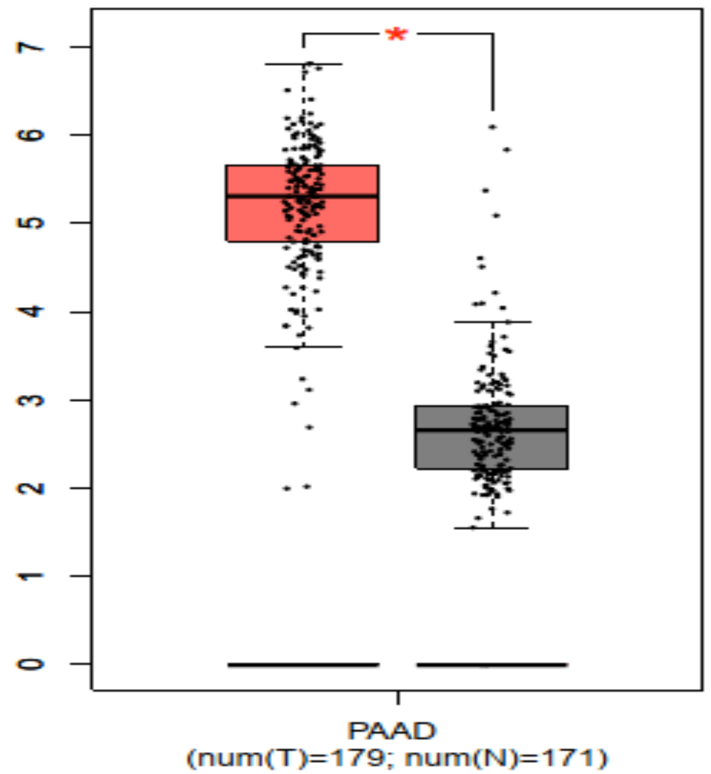

DBN1

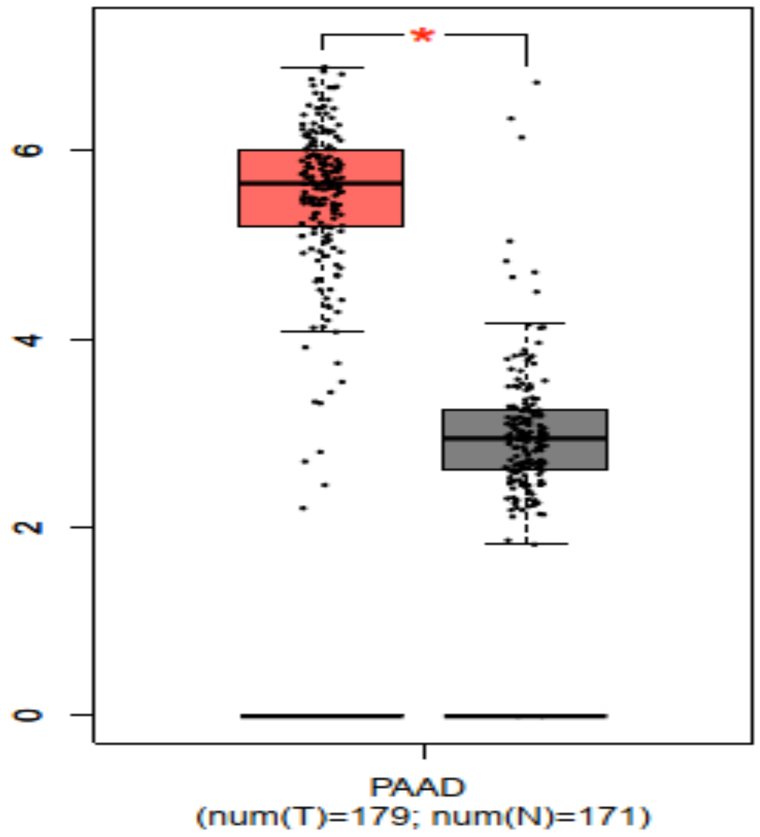

FLNA

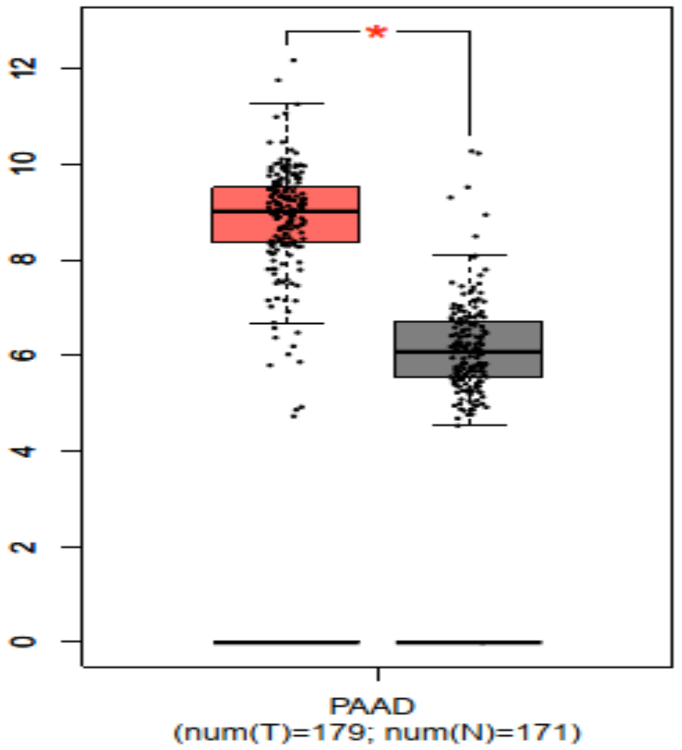
*
12
10
8
6
4
2
0
PAAD
(num(T)=179; num(N)=171)

GSN

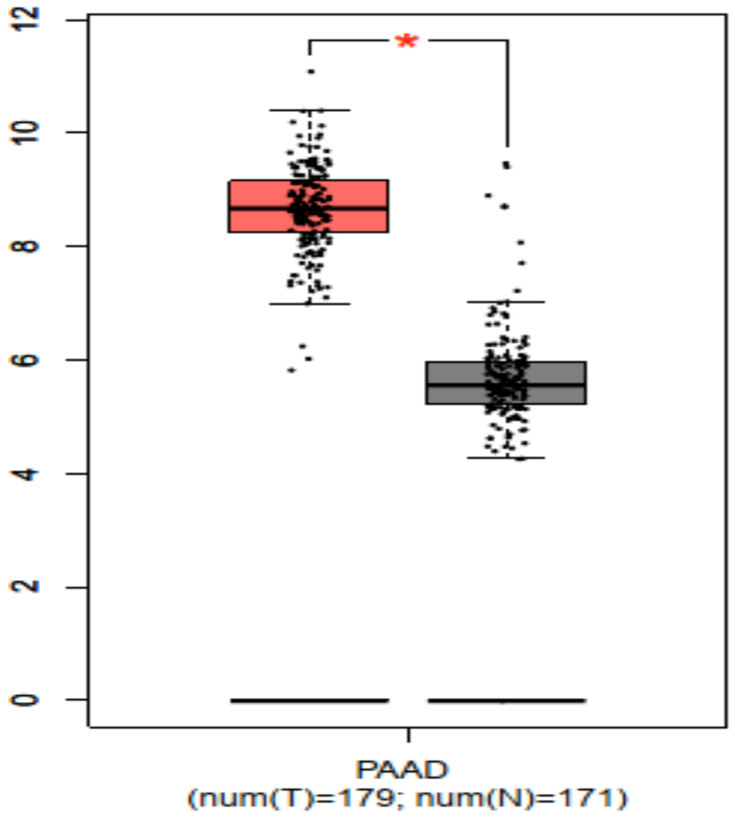
*
12
10
8
6
4
2
0
PAAD
(num(T)=179; num(N)=171)

IQGAP1

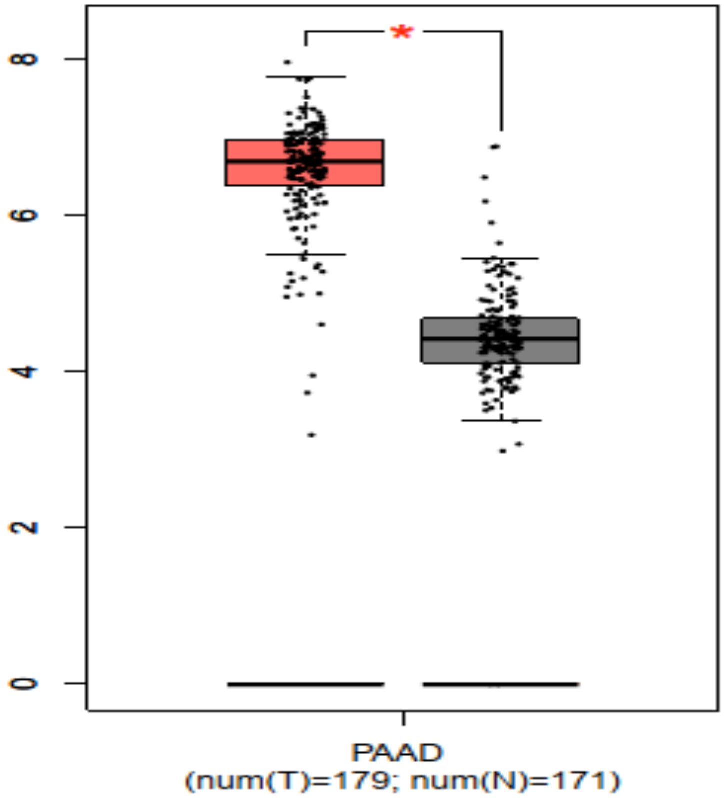

MYH9

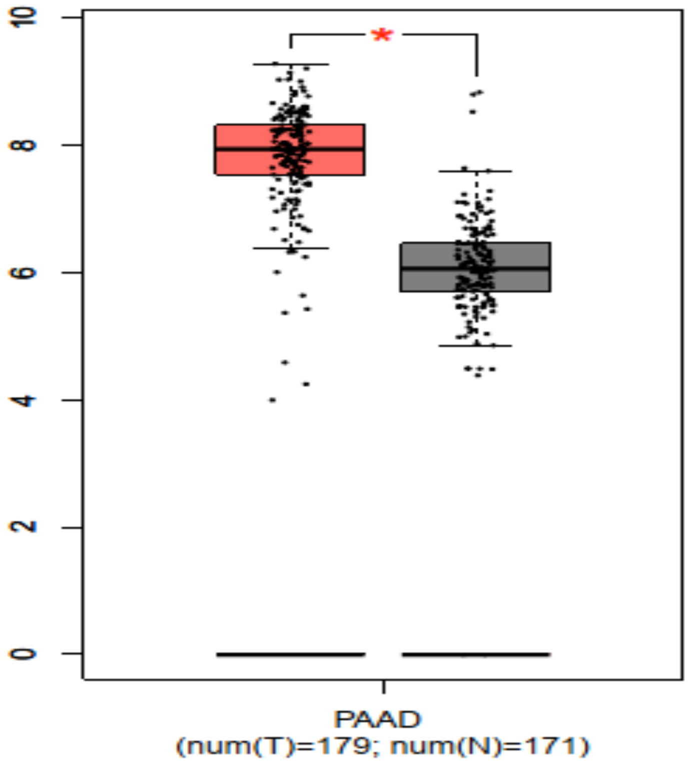

MYH10

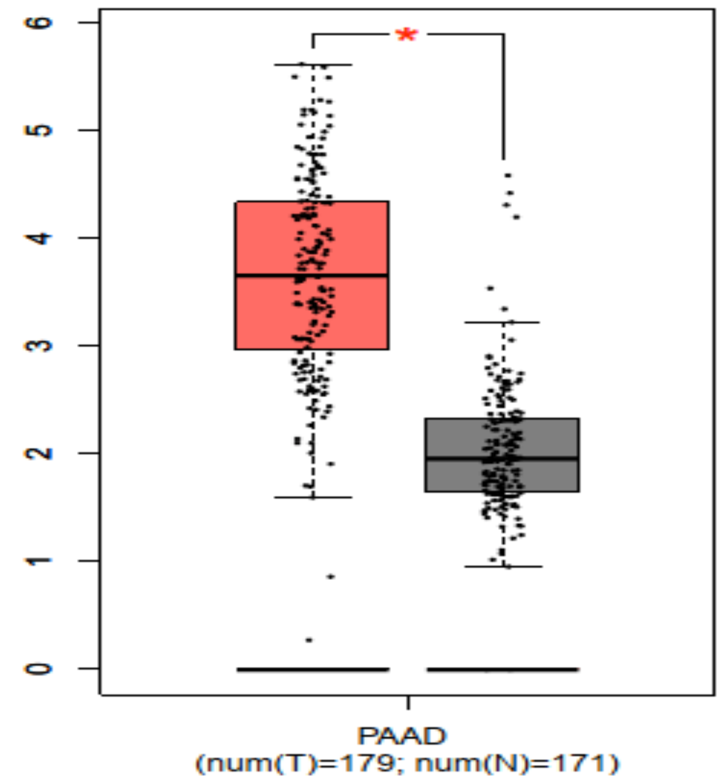
6
5
4
3
2
1
0
*
PAAD
(num(T)=179; num(N)=171)

MYO1C

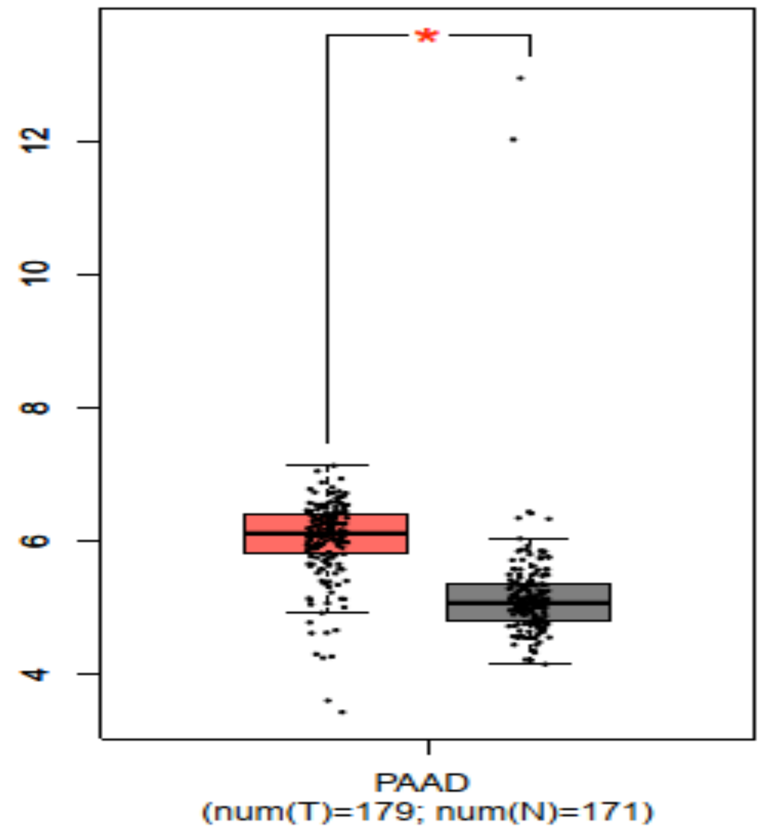
12
10
8
6
4
*
PAAD
(num(T)=179; num(N)=171)

MYO1E

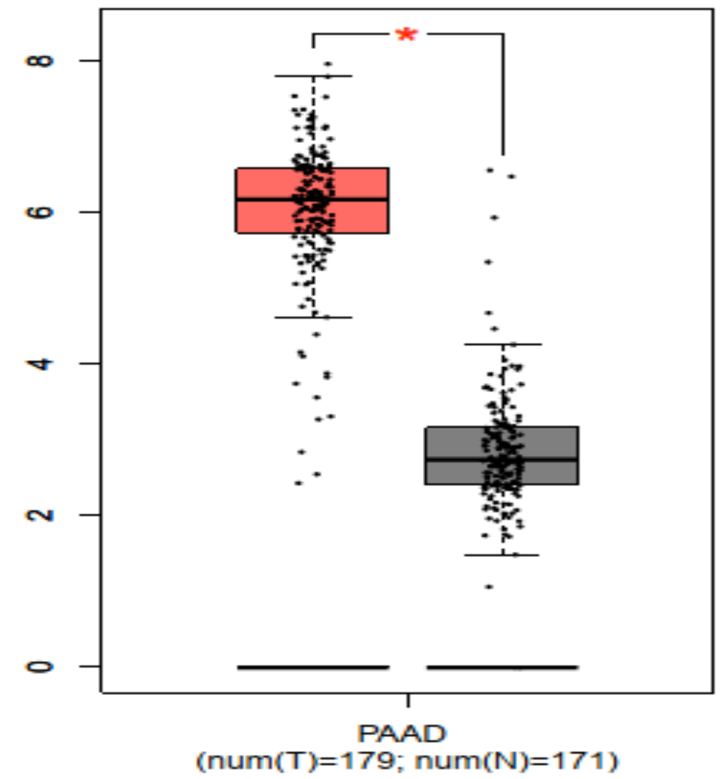

MYO5A

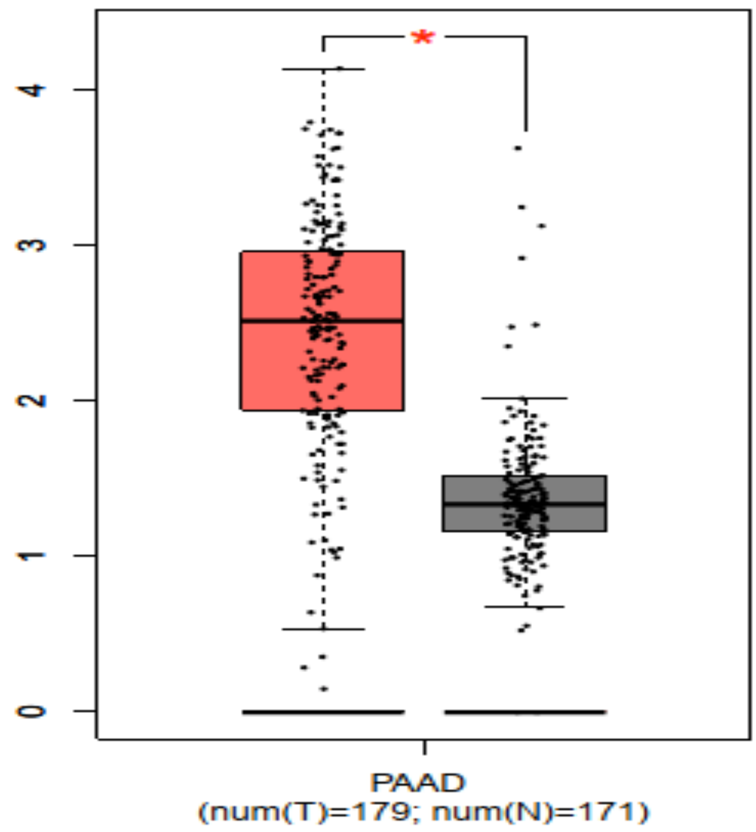

TPM1

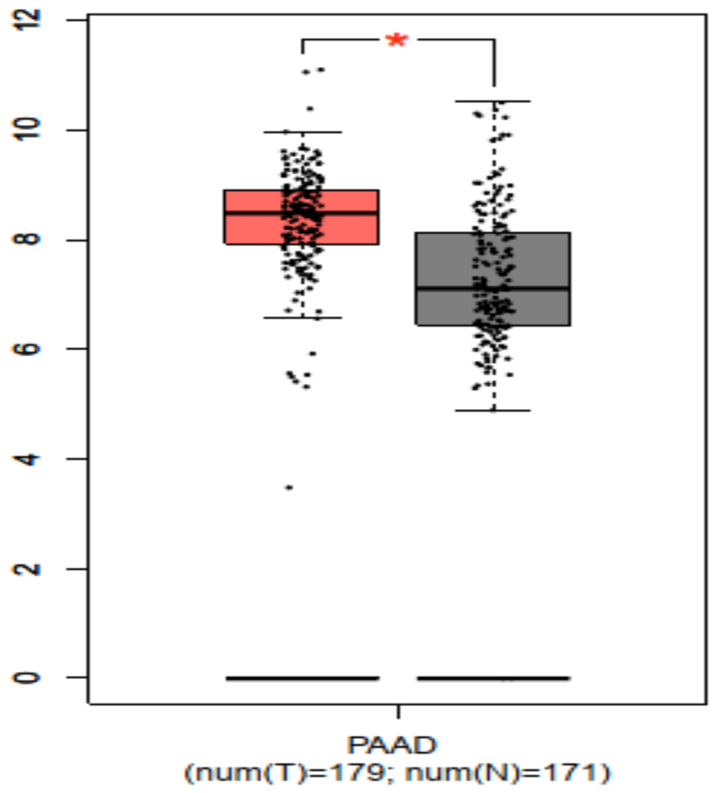
12
10
8
6
4
2
0
*
PAAD
(num(T)=179; num(N)=171)

TPM4

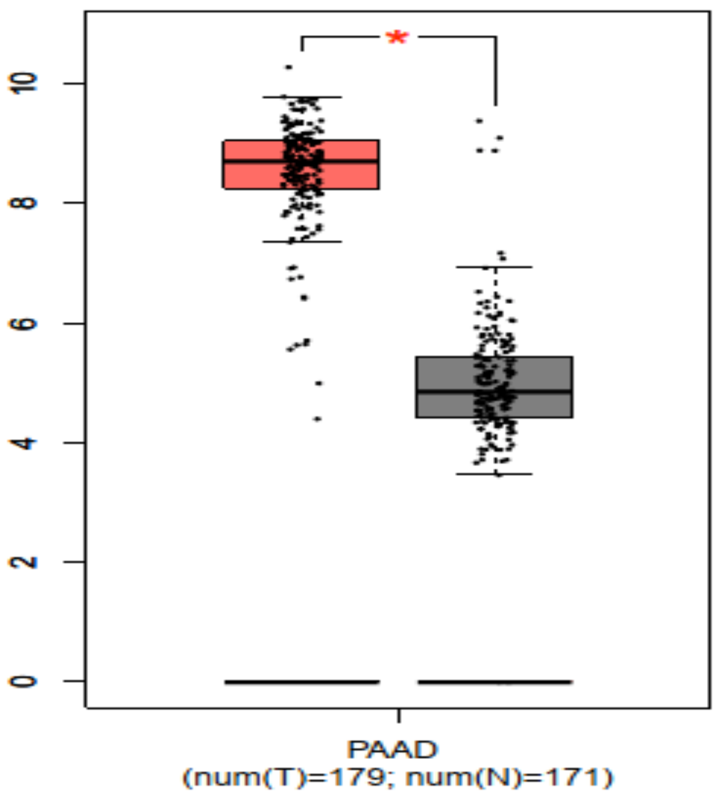
10
8
6
4
2
0
*
PAAD
(num(T)=179; num(N)=171)

WDR1

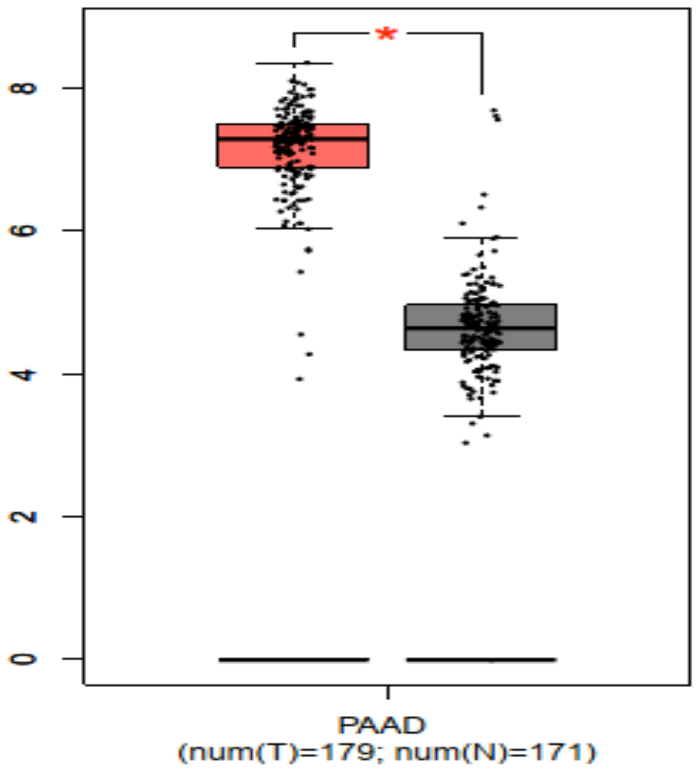
*
8
6
4
2
0
PAAD
(num(T)=179; num(N)=171)

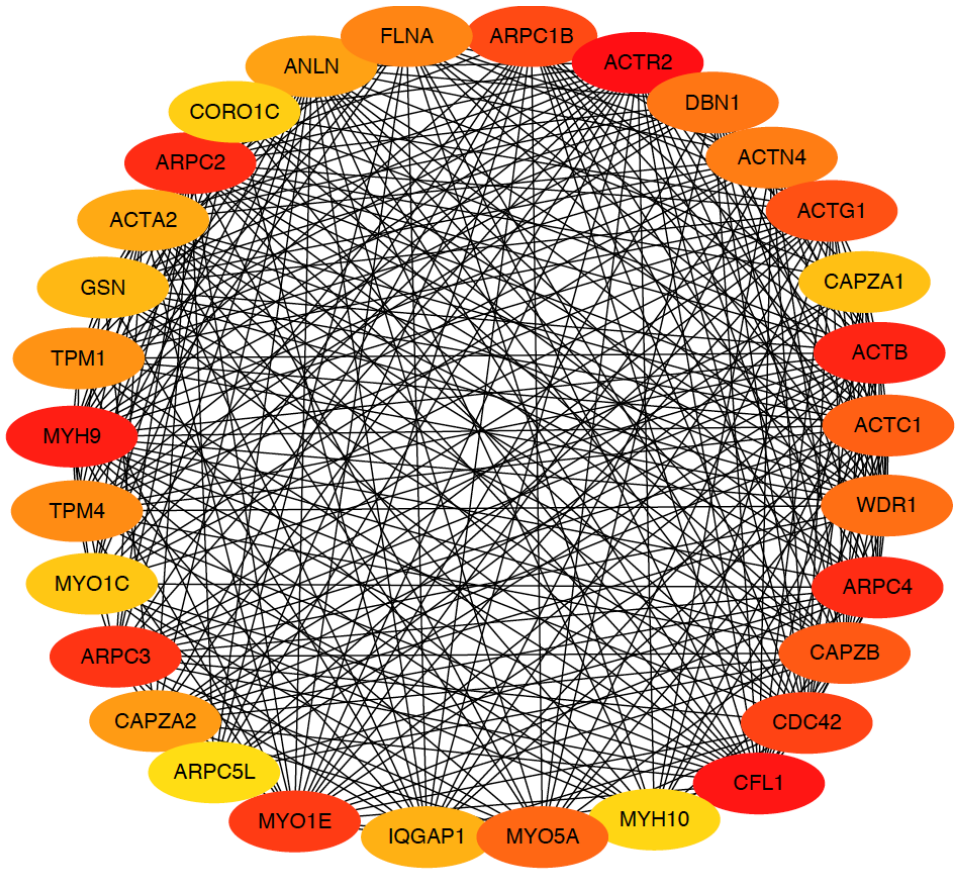
FLNA
ARPC1B
ANLN
ACTR2
DBN1
CORO1C
ARPC2
ACTN4
ACTA2
ACTG1
GSN
CAPZA1
TPM1
ACTB
MYH9
ACTC1
TPM4
WDR1
MYO1C
ARPC4
ARPC3
CAPZB
CAPZA2
CDC42
ARPC5L
CFL1
MYO1E
IQGAP1
MYO5A
MYH10
